# Individualized Treatment in Distal and Medium Vessel Occlusion Stroke Using a Validated Explainable Counterfactual Treatment Estimation Model

**DOI:** 10.1002/ana.78168

**Published:** 2026-01-28

**Authors:** Mohamed F. Doheim, Mahmoud H. Mohammaden, Hend Abdelhamid, Marta Olive‐Gadea, Marc Rodrigo‐Gisbert, Manuel Requena, Johanna T. Fifi, James E. Siegler, Santiago Ortega‐Gutierrez, Mohamad Abdalkader, Ali Alaraj, Wei Hu, Thanh N. Nguyen, Diogo C. Haussen, Raul G. Nogueira

**Affiliations:** ^1^ Departments of Neurology and Neurosurgery University of Pittsburgh Medical Center Pittsburgh PA USA; ^2^ Department of Neurology Emory University School of Medicine Atlanta GA USA; ^3^ Marcus Stroke and Neuroscience Center Grady Memorial Hospital Atlanta GA USA; ^4^ Department of Neurology Hospital Universitario Vall d'Hebron Barcelona Spain; ^5^ Department of Neurology Icahn School of Medicine at Mount Sinai New York NY USA; ^6^ Department of Neurology University of Chicago Chicago IL USA; ^7^ Department of Neurology University of Iowa Hospitals and Clinics Iowa City IA USA; ^8^ Department of Neurology Radiology, Boston University Chobanian and Avedisian School of Medicine Boston MA USA; ^9^ Department of Neurosurgery University of Illinois at Chicago Chicago IL USA; ^10^ Division of Life Sciences and Medicine, Department of Neurology The First Affiliated Hospital of USTC, University of Science and Technology of China Hefei China

## Abstract

**Objective:**

The optimal treatment for distal medium vessel occlusion (DMVO) stroke remains uncertain, and evidence comparing endovascular therapy (EVT) with medical management (MM) is limited. We aimed to develop and validate a predictive modeling tool to assess individual treatment benefit in DMVO stroke using explainable counterfactual treatment estimation.

**Methods:**

Adults with isolated DMVO stroke (M3–M4, A2–A3, or P1–P2) were retrospectively identified from 7 stroke centers. To estimate individualized probabilities of favorable outcome (modified Rankin Scale [mRS] = 0–2 at 90 days), we developed a Penalized Logistic Regression (Elastic Net) model. This framework was selected a priori over other explored machine learning algorithms (Decision Tree, Support Vector Classifier, and XGBoost) for its superior interpretability and ability to handle multicollinearity among interaction terms. Inverse Probability of Treatment Weighting (IPTW) was implemented to address confounding by indication in the observational data. Internal validation used repeated K‐fold cross‐validation and bootstrapping; external validation was performed on an independent cohort (n = 86).

**Results:**

Of 321 eligible patients, 179 received EVT (55.8%) and 142 received MM (44.2%). Adjusted models showed no significant overall group differences in favorable outcome (adjusted OR [aOR] = 1.32, 95% confidence interval [CI] = 0.97–1.80), mortality (aOR = 1.20, 95% CI = 0.78–1.85), or symptomatic hemorrhage (aOR = 0.57, 95% CI = 0.21–1.58). However, the model identified significant treatment effect heterogeneity; EVT benefit was amplified in patients with higher National Institutes of Health Stroke Scale (NIHSS) and attenuated with increasing treatment delay. Internal validation demonstrated strong performance (area under the receiver operating characteristic curve [AUC] = 0.77, 95% CI = 0.71–0.82). External validation confirmed generalizability (AUC = 0.74, 95% CI = 0.63–0.84). Individualized treatment estimates showed high concordance with a benchmark causal T‐Learner model (Pearson *r* = 0.97 internal and *r* = 0.98 external).

**Interpretation:**

Although aggregate outcomes did not differ significantly, the validated Distal and Medium Vessel Occlusion Stroke (DUSK) Tool enables individualized estimation of EVT benefit in DMVO stroke. This explainable counterfactual treatment estimation framework supports precision decision making by identifying specific patient subgroups most likely to benefit from EVT over MM. ANN NEUROL 2026;99:1198–1209

Acute ischemic stroke (AIS) remains a leading cause of disability and death worldwide, with clinical outcomes closely tied to the timeliness and appropriateness of therapeutic intervention.^1^, ^2^ Endovascular treatment (EVT) has become a cornerstone for managing large vessel occlusions (LVOs), supported by evidence from pivotal randomized controlled trials (RCTs) showing improved functional outcomes compared to medical management (MM).^3^, ^4^, ^5^, ^6^, ^7^ Meanwhile, distal and/or medium vessel occlusion (DMVOs) represent up to 40% of AIS.^8^, ^9^ Conservatively, they involve occlusions of smaller, more distal cerebral arteries, such as the M3/M4 branches of the middle cerebral artery (MCA), A2/A3 of the anterior cerebral artery (ACA), or P1/P2/P3 segments of the posterior cerebral artery (PCA), and represent a heterogeneous and increasingly recognized subgroup of AIS.^8^, ^9^, ^10^ Although often associated with milder symptoms at onset, DMVOs can nonetheless result in significant morbidity, particularly when eloquent territories are affected.^9^, ^10^

The clinical management of DMVOs poses a distinct challenge due to limited evidence supporting EVT use in this setting.^10^, ^11^, ^12^, ^13^, ^14^, ^15^ Recent RCTs have yielded inconclusive results, in part, due to potential selection biases such as the predominance of patients with mild to moderate strokes, an older patient population, a high rate of failed recanalization, and a high prevalence of intravenous thrombolysis (IVT) administration, the latter of which may be particularly effective in DMVOs.^15^, ^16^, ^17^, ^18^, ^19^ A recent meta‐analysis of DMVOs, including 38 studies (3 RCTs, 1 post hoc RCT analysis, 1 individual patient data meta‐analysis, and 33 observational studies) with 4,584 EVT‐treated and 5,468 BMT‐only patients, found that although EVT achieved high rates of successful recanalization (~78%), it did not confer functional outcome benefits over MM. Conversely, EVT was associated with increased risks of symptomatic intracranial hemorrhage, overall intracerebral hemorrhage (ICH), procedure‐related complications, and mortality.^20^ Consequently, the decision to proceed with EVT in DMVO cases is frequently based on physician discretion, local expertise, and institutional protocols, leading to significant variability in practice patterns and uncertainty in treatment benefit for individual patients.^9^, ^13^, ^19^, ^21^ This clinical dilemma urges the need for individualized decision‐support tools that can assist clinicians in navigating the nuanced risk–benefit landscape of DMVO treatment. Traditional risk stratification approaches or treatment guidelines are often insufficiently granular to accommodate the variability in anatomy, patient comorbidities, stroke severity, and time‐to‐treatment that characterizes the DMVO population.^10^, ^16^, ^17^, ^22^ In this context, predictive modeling offers a promising pathway toward precision medicine, leveraging data to tailor treatment recommendations to the profile of each patient.

The Distal and Medium Vessel Occlusion Stroke (DUSK) tool was developed in response to this clinical need as a data‐driven, clinically interpretable modeling framework. Unlike conventional outcome prediction models that estimate a single outcome probability, the DUSK tool uses counterfactual treatment estimation, predicting the likelihood of a favorable outcome under both EVT and MM scenarios for the same patient. By incorporating interaction terms and addressing treatment selection bias through established causal inference methodologies, the DUSK tool aims to bridge the gap between population‐level data and patient‐level decisions.

## Methods

### Standard Protocol Approvals, Registrations, and Patient Consents

This study was approved by the institutional review board (IRB). Due to the retrospective nature of the analysis, the IRB determined that informed consent from participants was not required and granted a waiver of consent. No identifiable images, videos, or other personal data requiring disclosure authorization were included in this manuscript.

### Patients and Settings

The patient population used to develop the DUSK tool was derived from a multicenter cohort of prospectively collected data from 7 comprehensive stroke centers (6 in the United States and 1 in Europe). Data collection spanned from January 1, 2017, to June 30, 2021. Each center followed local protocols for acute stroke imaging, treatment, and follow‐up, ensuring a high level of consistency in data quality. Data collection was approved by the IRB or Ethics Committee at each participating site. Given the retrospective nature of the current analysis and the use of fully anonymized data, the requirement for patient‐informed consent was waived. Anonymized data that support the findings of this study are available from the lead and corresponding authors, Mohamed F. Doheim and Raul G. Nogueira, upon reasonable request and following institutional data‐sharing policies.

The study included consecutive patients who presented with AIS due to isolated DMVOs. The inclusion and exclusion criteria were previously described.^10^ Patients were eligible for inclusion if they had a confirmed DMVO on baseline computed tomography angiography (CTA), with the occlusion located in the M3 or M4 segments of the MCA, A2, or A3 segments of the ACA, or the P1 or P2 segments of the PCA. Additionally, patients had a pre‐morbid modified Rankin Scale (mRS) score of 2 or less, a time from last known well (LKW) to imaging or treatment of 24 hours or less, and available 90‐day functional outcome data. Patients were excluded if they had a proximal LVO, multivessel occlusion, or missing data on core clinical variables such as pre‐stroke mRS. In addition, patients with PCA‐P3 occlusions were excluded due to uniform management with medical therapy, and those with ACA‐A1 occlusions were excluded due to inconsistencies in their anatomic classification.^8^, ^10^, ^16^, ^17^ Patients were categorized into 2 groups based on initial management: the EVT group, who underwent thrombectomy, and the MM group. Eligible patients received IVT with alteplase if within the treatment window and without contraindications. EVT decisions were made by local treating teams without prespecified criteria, reflecting real‐world practice and necessitating the use of propensity‐score weighting to address potential confounding by indication. This study was conducted and reported in accordance with the Transparent Reporting of a multivariable prediction model for Individual Prognosis or Diagnosis (TRIPOD) guidelines.^23^

### Data Preparation and Feature Engineering

**FIGURE 1:**
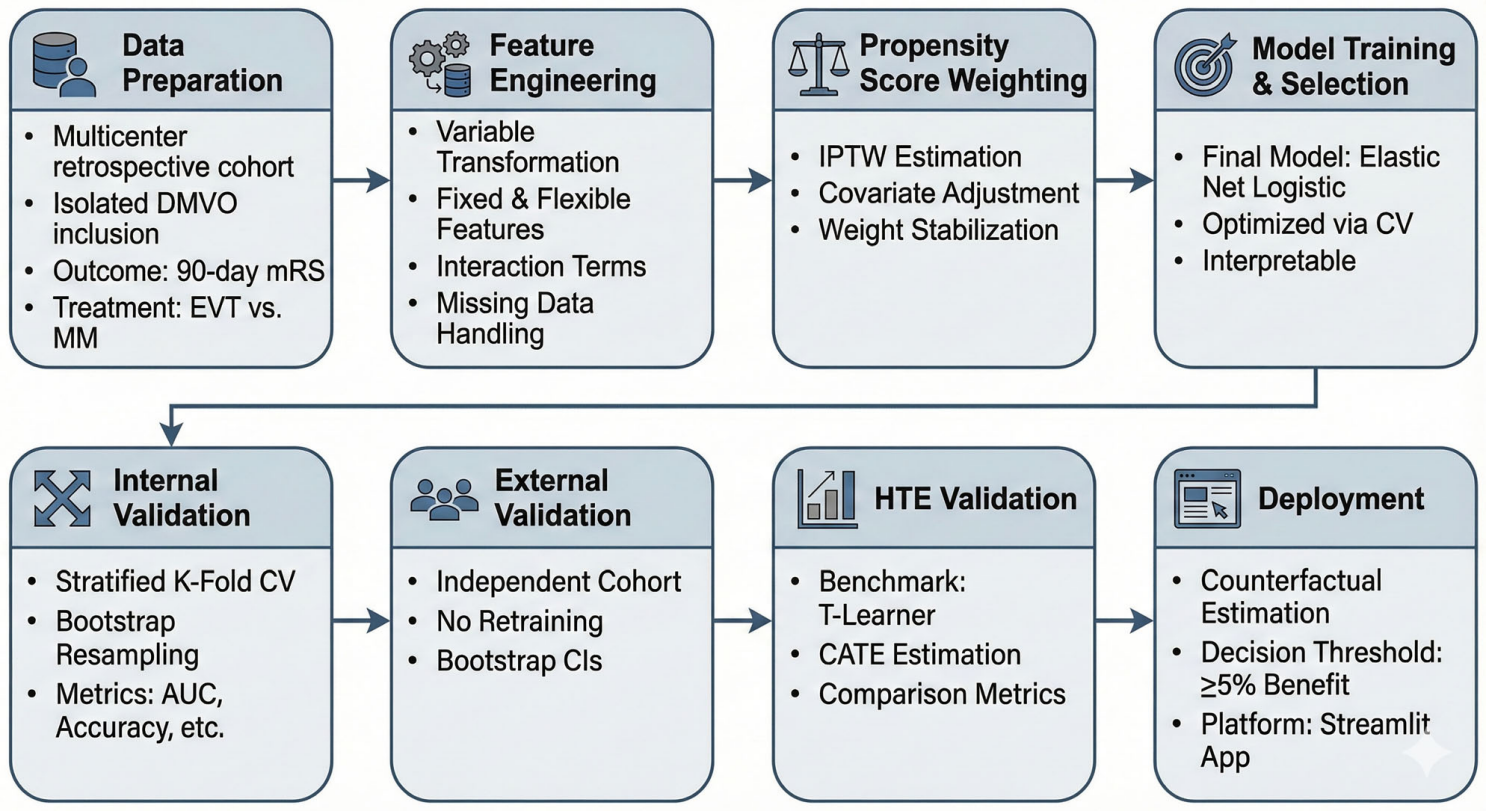
Development and validation pipeline of the DUSK clinical decision support tool. The workflow illustrates the sequential phases of the study: (1–2) Data Preparation and Engineering: Utilization of a multicenter retrospective cohort of patients with isolated DMVO, followed by variable transformation, missing data imputation, and the generation of fixed, flexible, and interaction features. (3) Bias Adjustment: Implementation of IPTW to balance covariates between treatment groups. (4) Modeling: Training and selection of an Elastic Net Logistic Regression model optimized via cross‐validation. (5–7) Validation: Rigorous performance assessment using internal stratified k‐fold cross‐validation, external validation on an independent cohort, and HTE benchmarking against a T‐Learner meta‐learner. (8) Deployment: Final implementation of the model into a web‐based interface (Streamlit) to provide counterfactual probability estimates for EVT versus MM. DMVO = distal medium vessel occlusion; DUSK = distal and medium vessel occlusion stroke; EVT = endovascular therapy; HTE = heterogeneity of treatment effect; IPTW = inverse probability of treatment weighting; MM = medical management. [Color figure can be viewed at www.annalsofneurology.org]

Analyses were performed using Python (version 3.13), with workflow steps summarized in Figure 1. The dataset underwent rigorous preprocessing for consistency and optimization. Key variables included the treatment indicator and a binary outcome of favorable functional status, defined as a 90‐day mRS score of 0–2. This threshold was selected specifically to achieve class balance distribution during model training and to accurately include return‐to‐baseline outcomes for patients with a pre‐stroke mRS of 2.^24^ Continuous variables, including age, National Institutes of Health Stroke Scale (NIHSS), glucose, and time to treatment, were converted to numeric types, whereas relevant categorical and binary factors were binary‐coded. Feature engineering involved creating clinically meaningful binary predictors based on established literature and domain expertise: age > 80 years, NIHSS ≥ 8, glucose > 180 mg/dL, baseline disability (mRS ≤ 1 vs. 2), treatment time within 360 minutes, and site of occlusion (anterior vs. posterior) based on previous domain knowledge from experts and literature.^10^, ^14^, ^17^, ^18^, ^25^, ^26^, ^27^, ^28^ o avoid collinearity, a combined variable was generated to indicate the presence of either hyperlipidemia or a smoking history.^29^, ^30^ Variables with >10% missingness, such as CTP‐based metrics, including core volume, mismatch volume, and Tmax thresholds, were excluded from model development. Remaining missing data were imputed using multiple imputations with 10‐fold chained equations to preserve data integrity and reduce bias.^31^, ^32^

### Statistical Modeling and Internal Validation

To estimate the probability of a favorable outcome and personalize recommendations, we initially evaluated four algorithms: Logistic Regression, Decision Tree, Support Vector Classifier, and XGBoost. The Penalized Logistic Regression (Elastic Net) model was selected a priori as the final framework based on its methodological robustness. Unlike automated stepwise selection, which often yields biased estimates and is prone to overfitting, Elastic Net utilizes L1 (Lasso) and L2 (Ridge) penalties to encourage sparsity and handle multicollinearity. Regularization hyperparameters were optimized via 5‐fold cross‐validation.^33^, ^34^ To address treatment selection bias, Inverse Probability of Treatment Weighting (IPTW) was implemented. Propensity scores were estimated using a logistic model including all core clinical covariates; weights were stabilized and truncated at the first and 99th percentiles, achieving excellent covariate balance (standardized mean differences = < 0.1). These weights were applied during coefficient estimation but are not used for individual patient predictions.^35^ The final model incorporated interaction terms between treatment and clinical variables (age, disability, NIHSS, site, time, and thrombolysis) to capture treatment effect modification. Model performance was rigorously assessed using repeated K‐fold cross‐validation (10 folds and 500 repetitions) and 1,000 bootstrap iterations to derive 95% confidence intervals (CIs) for area under the receiver operating characteristic curve (AUC), Brier score, sensitivity, and accuracy.^36^, ^37^

### External Validation

The model was externally validated on an independent dataset (n = 86) from a large health care network using the pretrained model without retraining. Performance was evaluated via AUC, calibration plots, and classification metrics, with 95% CIs derived from 1,000 bootstrap iterations.^23^ We further benchmarked estimates against a causal T‐Learner approach to confirm agreement in heterogeneous treatment effect estimation.^38^

### Model Deployment

The DUSK tool is deployed as a high‐performance Streamlit web application, utilizing the Penalized Logistic Regression (Elastic Net) model and preprocessing metadata via joblib with caching for performance optimization. The application replicates the training pipeline by binarizing continuous variables, encoding categorical data, and dynamically generating treatment interaction terms to ensure consistent input handling. The deployment performs counterfactual treatment estimation by simultaneously predicting probabilities of favorable outcome for both EVT and MM scenarios for each patient. These probabilities are then transformed into an intuitive DUSK Clinical Score (ranging from 1 to 20) alongside raw log‐odds and odds ratios. To guide clinical decision making, a predefined ±5% differential benefit threshold is utilized to recommend one management strategy over the other. The Streamlit interface collects and validates real‐time patient data via sidebar widgets, delivering interpretable guidance and interactive visualizations to support transparency in the decision‐making process. Finally, the pipeline incorporates automated error handling and logging to ensure system robustness and reliability in clinical settings.

## Results

### Cohort Characteristics and Outcomes

Of 935 screened patients with DMVO, 321 met eligibility criteria; 179 (55.8%) received EVT and 142 (44.2%) received MM. EVT‐treated patients were older (mean age = 69.4 ± 13.0 vs. 66.4 ± 13.5 years), had higher baseline NIHSS scores (median 10, interquartile range [IQR] = 7–16 vs. 6, IQR = 3–11), and longer time from last known normal to imaging/treatment (median 340, IQR = 200–694 vs. 249, IQR = 120–629 minutes). The distribution of raw mRS scores at 90 days for patients treated with EVT and MM is illustrated in Supplementary Figure S1. Adjusted models showed no statistically significant differences between EVT and MM for good outcome (mRS = 0–2, aOR = 1.32, 95% CI = 0.97–1.80), excellent outcome (mRS = 0–1, aOR = 1.32, 95% CI = 0.94–1.85), or 90‐day mortality (aOR = 1.20, 95% CI = 0.78–1.85). Additionally, the multivariable regression analysis showed no significant difference in the rates of symptomatic intracranial hemorrhage between the EVT and MM groups (aOR = 0.57, 95% CI = 0.21–1.58, *p* = 0.277).

### Model Development, Internal Validation Performance, and Final Model Selection

**FIGURE 2:**
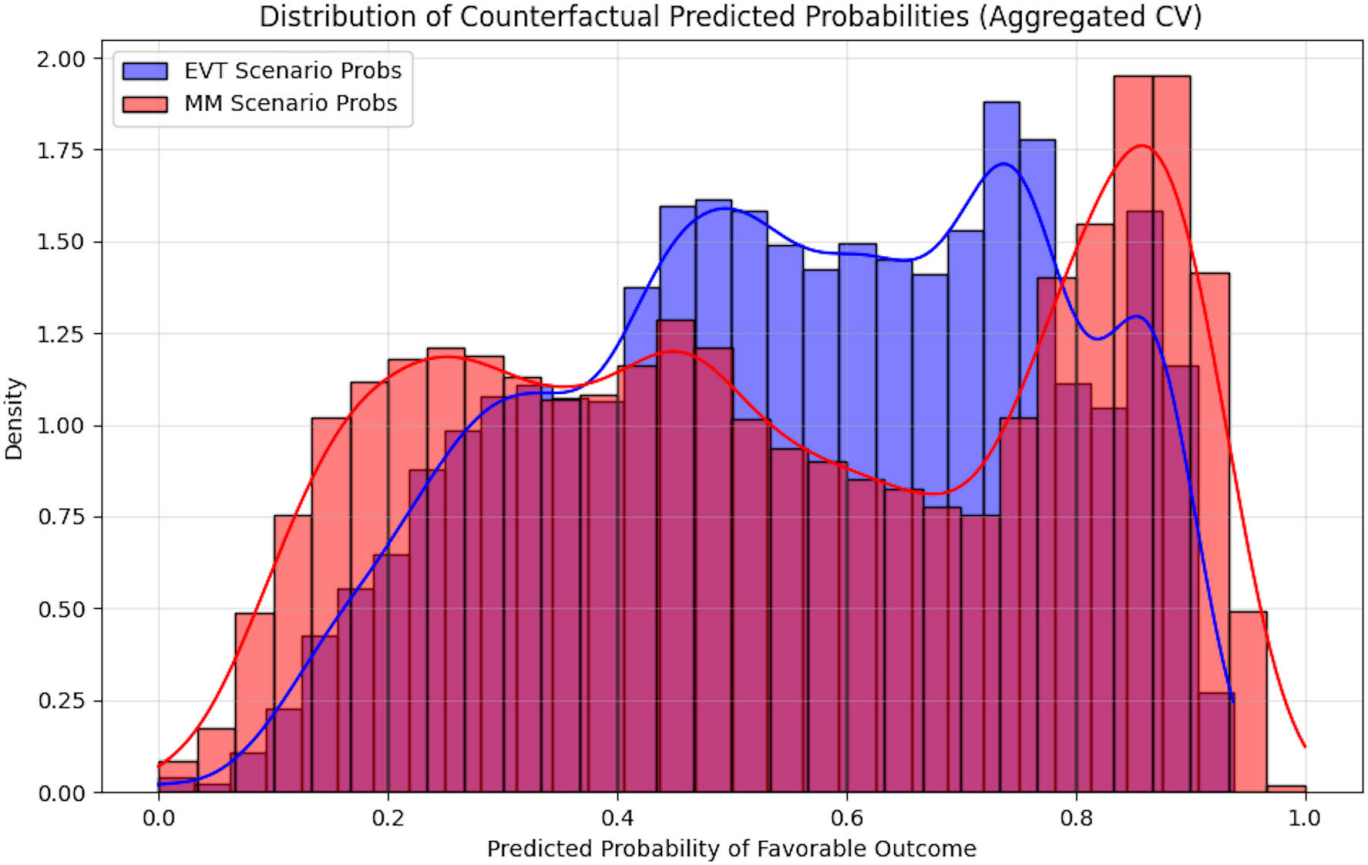
Distribution of counterfactual predicted probabilities (internal validation). Overlapping density histograms showing the distribution of predicted probabilities of favorable outcome under 2 treatment scenarios: EVT (*blue*) and MM (*red*). For each patient in the development cohort, the model estimates the probability of favorable outcome if treated with EVT versus if treated with MM, using counterfactual estimation. The substantial overlap indicates heterogeneity in predicted treatment benefit across the population. The separation between distributions at higher probability ranges suggests a subgroup of patients with meaningful differential EVT benefit. Kernel density estimation curves are superimposed to illustrate the continuous distribution shape. EVT = endovascular therapy; MM = medical management. [Color figure can be viewed at www.annalsofneurology.org]

**FIGURE 3:**
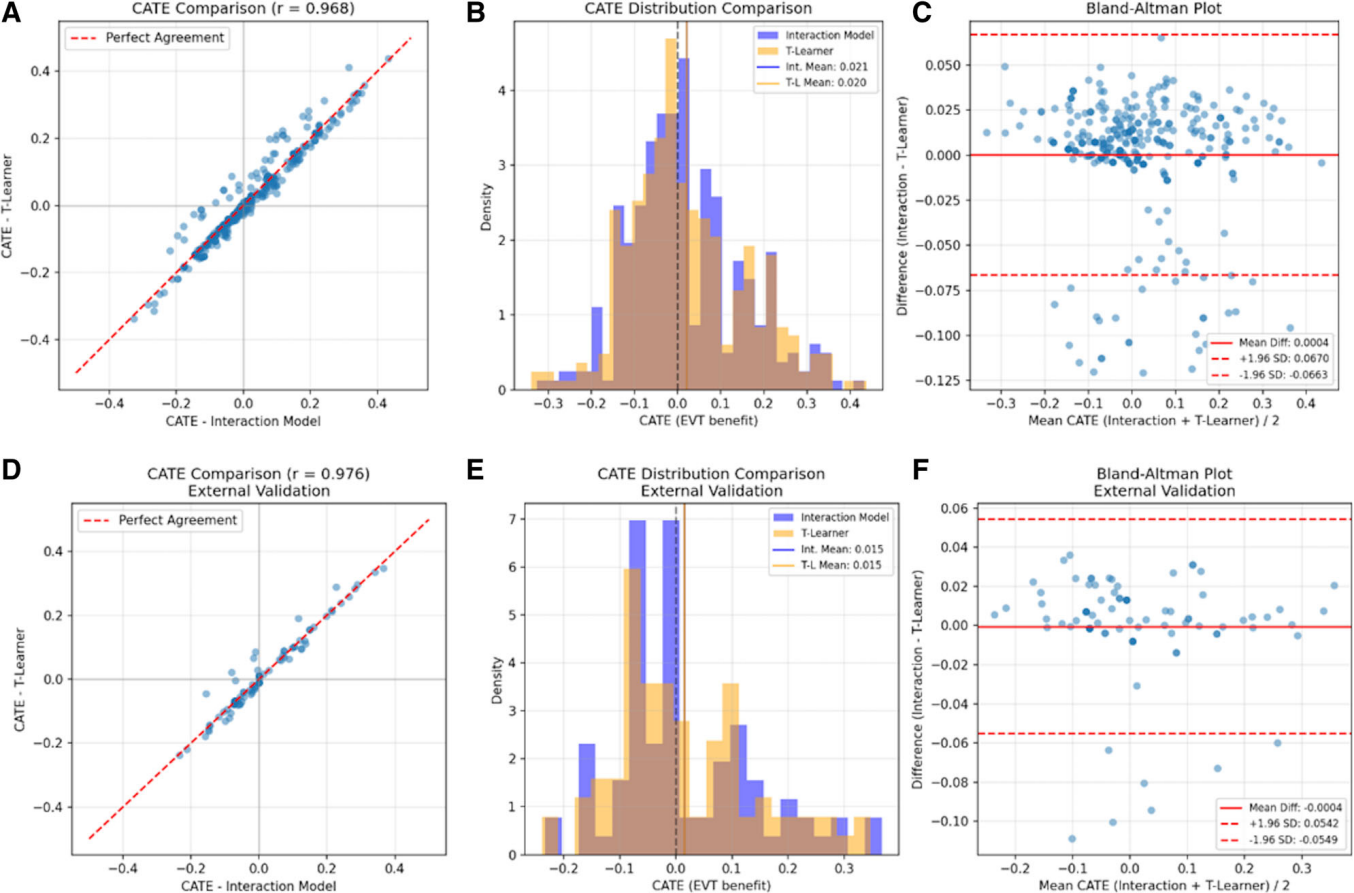
CATE comparison – interaction model versus T‐learner (internal and external validation). Six‐panel comparison validating heterogeneity of treatment effect estimates across internal and external cohorts. Internal Validation (A–C): (A) Scatter plot of CATE estimates from the interpretable Interaction Model (x‐axis) versus the causal T‐Learner benchmark (y‐axis), showing excellent agreement (Pearson *r* = 0.968; *red dashed line* = perfect concordance). (B) Overlapping histograms of CATE distributions (*blue* = interaction model, mean = 0.021; *orange* = T‐Learner, mean = 0.020), confirming similar central tendency and spread. (C) Bland–Altman plot illustrating minimal bias (mean difference = 0.0004) and narrow 95% limits of agreement (±0.067), supporting consistency across the range of estimates. External Validation (D–F): (D) Scatter plot demonstrating even stronger correlation (*r* = 0.976) between models in the external cohort. (E) Distribution comparison showing near‐identical mean CATE values (Interaction = 0.015; T‐Learner = 0.015) and variance. (F) Bland–Altman plot with negligible bias (mean difference = −0.0004) and tight 95% limits of agreement (±0.055), confirming robust generalizability of treatment effect heterogeneity estimation. CATE = conditional average treatment effect. [Color figure can be viewed at www.annalsofneurology.org]

The Penalized Logistic Regression (Elastic Net) model was selected as the final engine for the DUSK tool based on its superior stability and interpretability during comparative evaluations, outperforming alternative algorithms, such as Decision Tree, Support Vector Classifier, and XGBoost (Supplementary Table S1). The model integrates core predictors, including age, NIHSS, pre‐stroke disability, and occlusion site, alongside treatment‐covariate interaction terms to estimate the Conditional Average Treatment Effect (CATE; Table 1 and Supplementary Fig S2). Key coefficients indicated that higher baseline NIHSS (aOR = 0.35, 95% CI = 0.22–0.55) and treatment delays were strongly associated with reduced odds of favorable outcome. Although individual interaction *p* values were penalized and not all reached statistical significance, their inclusion enabled the identification of clinically relevant subgroups where EVT benefit was either amplified (eg, higher NIHSS) or attenuated (eg, treatment delay; see Table 1 and Supplementary Fig S2). Internal validation using repeated stratified 10‐fold cross‐validation and 1,000 bootstrap iterations demonstrated robust discriminative power and calibration, yielding an average AUC of 0.77 (95% CI = 0.71–0.82), a Brier score of 0.19 (95% CI = 0.17–0.21), a sensitivity of 77.8%, and an overall accuracy of 71.6%. Counterfactual predicted probability distributions confirmed significant heterogeneity in treatment benefit across the population (Fig 2 and Table 2). Furthermore, benchmarking against a sophisticated causal T‐Learner model revealed high concordance in treatment recommendations (92.5% agreement) and an extremely strong correlation in estimated benefit (Pearson *r* = 0.968), reinforcing the reliability of the individualized treatment estimation framework (Fig 3 and Supplementary Table S2).

**TABLE 1:** Multivariable Penalized Logistic Regression Model (Elastic Net) for Favorable Outcome

Predictor Variable	Coefficient	95% CI (Coef)	Adjusted OR	95% CI (OR)	*p*
Main effects					
NIHSS ≥ 8	−1.052	−1.51 to −0.60	0.349	0.22–0.55	< 0.001
Pre‐stroke mRS 0–1	0.247	−0.02 to 0.51	1.280	0.98–1.67	0.067
Tandem lesion	−0.222	−0.50 to 0.05	0.801	0.61–1.05	0.114
Atrial fibrillation	−0.296	−0.71 to 0.12	0.743	0.49–1.13	0.164
Diabetes mellitus	−0.280	−0.72 to 0.16	0.756	0.49–1.17	0.207
Anterior circulation	0.259	−0.18 to 0.70	1.296	0.84–2.01	0.246
Time >6 h	0.244	−0.23 to 0.72	1.276	0.79–2.06	0.318
Glucose > 180 mg/dl	−0.115	−0.40 to 0.17	0.891	0.67–1.19	0.431
IVT	0.126	−0.34 to 0.60	1.134	0.71–1.82	0.599
HLD/smoking history	0.102	−0.30 to 0.51	1.107	0.74–1.66	0.622
Age > 80 y	−0.113	−0.57 to 0.34	0.893	0.57–1.40	0.623
Male sex	0.016	−0.25 to 0.28	1.016	0.78–1.33	0.907
Intercept	0.339	0.09 to 0.59	1.403	1.09–1.81	0.009
Interaction terms, treatment modifiers					
Treatment, EVT	−0.071	−0.71 to 0.57	0.931	0.49–1.77	0.828
Treatment × NIHSS ≥ 8	0.406	−0.16 to 0.97	1.501	0.85–2.64	0.159
Treatment × atrial fibrillation	0.346	−0.10 to 0.80	1.413	0.90–2.22	0.133
Treatment × time >6 h	−0.290	−0.85 to 0.27	0.749	0.43–1.31	0.314
Treatment × anterior	0.104	−0.40 to 0.61	1.109	0.67–1.84	0.687
Treatment × age > 80 y	−0.122	−0.59 to 0.35	0.885	0.55–1.41	0.609
Treatment × HLD/smoking	−0.145	−0.63 to 0.34	0.865	0.53–1.41	0.559
Treatment × diabetes	−0.019	−0.45 to 0.41	0.981	0.64–1.51	0.930
Treatment × i.v. tPA	0.015	−0.51 to 0.54	1.015	0.60–1.71	0.955

*Note*: Main Effects represent the independent impact of clinical variables on outcome. Interaction Terms modify the effect of EVT relative to Medical Management. Whereas specific interaction *p* values may not reach statistical significance individually due to penalization, their inclusion allows the model to estimate the Conditional Average Treatment Effect (CATE), identifying subgroups with differential treatment benefit.

Abbreviations: CI = confidence interval; EVT = endovascular therapy; HLD = hyperlipidemia; IVT = intravenous thrombolysis; mRS = modified Rankin Scale; NIHSS = National Institutes of Health Stroke Scale; OR = odds ratio; tPA = tissue plasminogen activator.

**TABLE 2:** Internal Validation Performance (Repeated Stratified K‐Fold Cross‐Validation with Bootstrap)

Performance Metric	Mean Value	Standard Deviation	95% Confidence Interval
AUC, discrimination	0.768	0.027	0.714–0.820
Brier score, calibration	0.192	0.011	0.171–0.213
Accuracy	71.6%	2.5%	66.7–76.3%
Sensitivity	77.8%	3.1%	71.5–83.9%
Specificity	63.4%	4.2%	54.6–71.1%
Precision, PPV	73.5%	3.2%	67.4–79.8%
NPV	68.6%	4.2%	60.3–77.0%
F1 score	0.756	0.025	0.707–0.801

*Note*: Results based on 5‐fold cross‐validation with 100 repetitions (500 total folds) and 1,000 bootstrap iterations. The model demonstrates good discrimination (AUC = 0.77) and high sensitivity (77.8%), prioritizing identification of patients likely to have a favorable outcome.

Abbreviations: AUC = area under the receiver operating characteristic curve; NPV = negative predictive value; PPV = positive predictive value.

### External Validation

**FIGURE 4:**
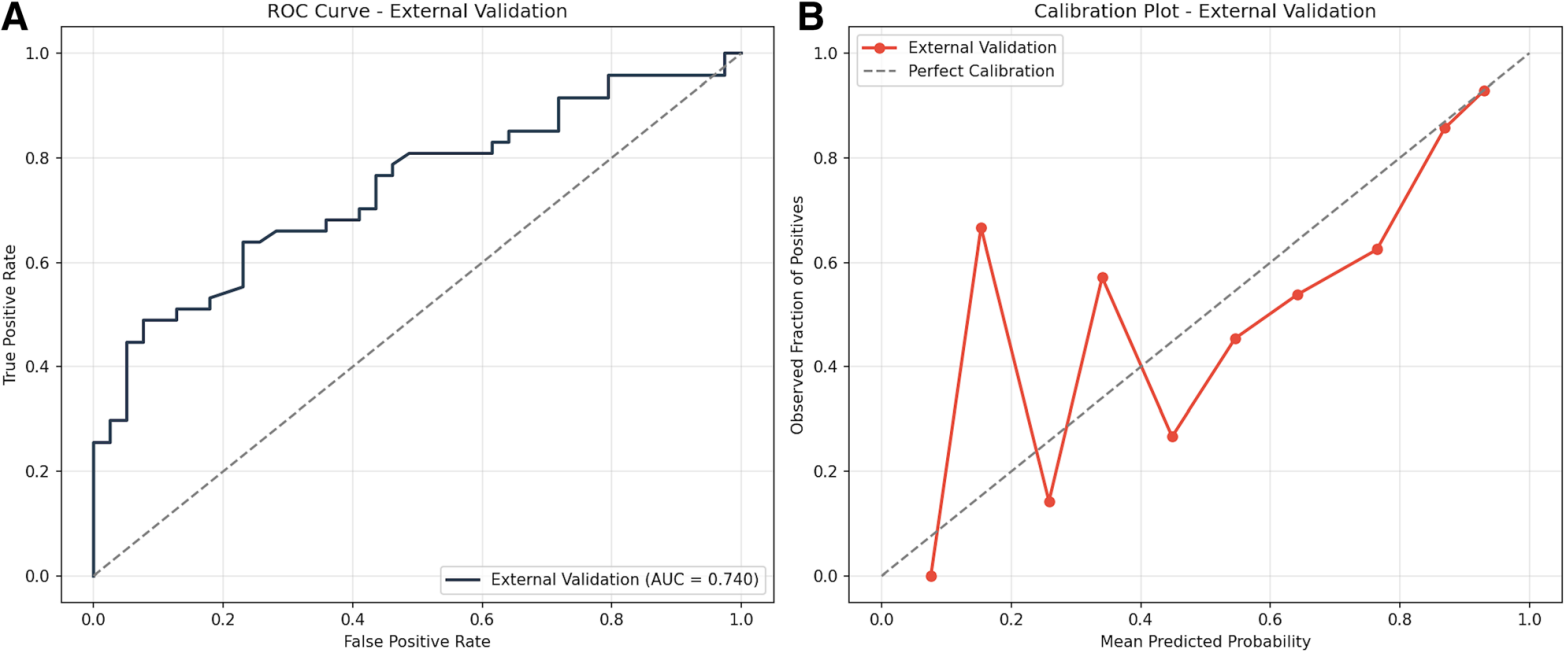
External validation performance – ROC and calibration. (A) ROC curve for external validation demonstrating maintained discrimination in an independent cohort. AUC = 0.740 (95% CI = 0.628–0.835). The slight decrease from internal validation (AUC = 0.77) is expected and indicates appropriate model generalizability without substantial overfitting. (B) Calibration Plot showing observed versus predicted probabilities in the external cohort. Whereas calibration shows more variability than internal validation (expected with smaller sample size, n = 86), the overall trajectory follows the diagonal, indicating reasonable probability estimation in new clinical settings. AUC = area under the receiver operating characteristic curve; CI = confidence interval; ROC = receiver operating characteristic. [Color figure can be viewed at www.annalsofneurology.org]

To assess generalizability, the final model was externally validated using an independent cohort of 86 patients (EVT, n = 49; MM, n = 37; Supplementary Table S3). The median age was 72.0 years (IQR = 61.0–78.8 years), with no significant age difference between groups (*p* = 0.164). The treatment group had a higher median NIHSS score (13.0, IQR = 10.0–20.0) compared with controls (6.0, IQR = 4.0–10.0, *p* < 0.001). Other baseline characteristics, including diabetes mellitus, atrial fibrillation, gender distribution, and glucose levels, were similar between groups. Time metrics differed significantly, with longer median times observed in the treatment group (280 vs. 203 minutes, *p* = 0.033). Performance metrics derived from 1,000 bootstrap samples confirmed robust generalizability, with an average AUC of 0.74 (95% CI = 0.63–0.84) and a Brier score of 0.21 (95% CI = 0.17–0.25). Other validation metrics remained consistent, including a sensitivity of 76.6% and an overall accuracy of 67.5% (Supplementary Table S4 and see Fig 3, 4). Benchmarking against a causal T‐Learner model demonstrated exceptional external reliability, maintaining an extremely strong correlation in treatment benefit estimates (Pearson *r* = 0.98) and high concordance in clinical recommendations (89.5% agreement; see Fig 3 and Supplementary Table S4).

### Application of the DUSK Tool in Clinical Practice

**FIGURE 5:**
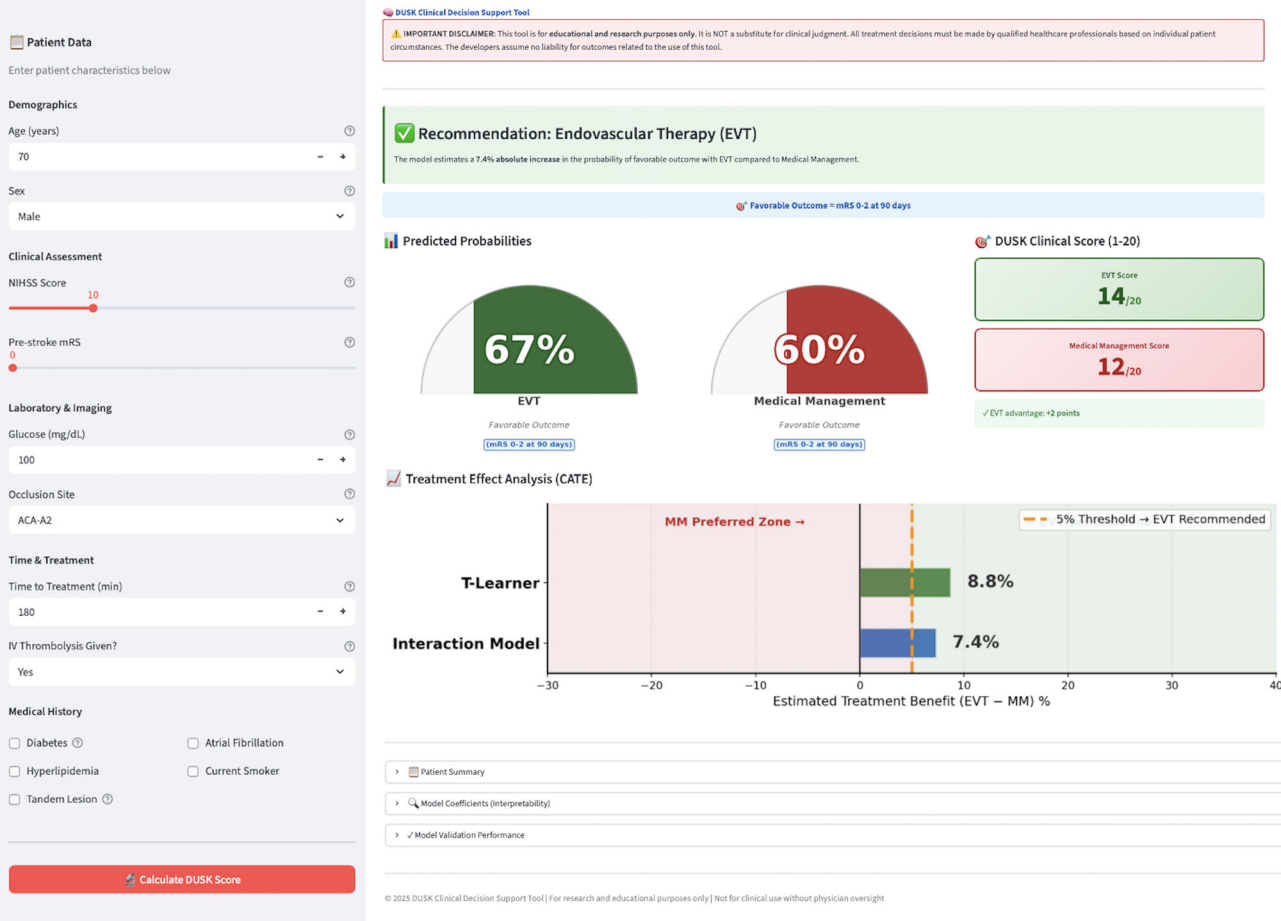
Web application interface – DUSK tool. Snapshot of the deployed DUSK tool web application (https://dusktool.streamlit.app/). The interface allows clinicians to input patient‐specific variables including age, NIHSS score, time from symptom onset, occlusion site (anterior/posterior), pre‐stroke mRS, glucose level, and relevant comorbidities. The tool displays: (1) predicted probability of favorable outcome under EVT scenario; (2) predicted probability of favorable outcome under the MM scenario; (3) estimated differential treatment benefit; and (4) treatment recommendation based on a 5% benefit threshold. The output emphasizes that predictions are estimates from observational data and should be integrated with clinical judgment rather than used as sole decision criteria. DUSK = Distal and Medium Vessel Occlusion Stroke; EVT = endovascular therapy; MM = Medical Management; mRS = modified Rankin scale; NIHSS = National Institutes of Health Stroke Scale. [Color figure can be viewed at www.annalsofneurology.org]

Because the forward selection model demonstrated slightly superior performance on internal and external validation, it was selected for deployment. A web‐based application was developed (https://dusktool.streamlit.app/) to facilitate real‐time clinical use of the model (Figs 4 and 5). The tool allows entry of individual patient characteristics and provides personalized treatment estimation using counterfactual treatment estimation. The tool enables clinicians to input patient‐specific characteristics, including demographics, NIHSS, pre‐stroke disability, laboratory values, occlusion site, time to treatment, and comorbidities, and returns individualized predictions of favorable outcome under both EVT and MM. Outputs include predicted probabilities, an interpretable DUSK Clinical Score (range = 1–20), and a treatment recommendation based on a predefined ±5% threshold for clinically meaningful benefit. Additionally, the interface visualizes treatment effect heterogeneity by displaying CATE estimates from both the interpretable interaction model and the causal T‐Learner benchmark, supporting transparency and confidence in decision making.

## Discussion

This study introduces the DUSK tool, a novel counterfactual modeling framework designed to guide individualized treatment decisions for patients with isolated DMVO stroke by simulating patient outcomes under both EVT and MM alone. The DUSK tool estimates the differential benefit of EVT based on each patient's clinical and imaging profile. Built using transparent logistic regression with curated domain‐specific features and clinically meaningful interaction terms, the tool emphasizes both interpretability and precision. In doing so, the DUSK tool bridges the gap between population‐level data and patient‐level decisions, addressing a major evidence gap in DMVO management where randomized trial data remains limited and inconclusive. Despite a high technical success rate of EVT (successful reperfusion in 89.4% of cases), our unstratified but adjusted analyses demonstrated no statistically significant difference between EVT and MM across primary and secondary functional outcomes including the 90‐day ordinal mRS shift, mRS 0–2 (“good outcome”), mRS 0–1 (“excellent outcome”), or mortality.^10^ These findings mirror prior observational studies and even RCTs, underscoring the limitations of traditional binary comparisons in a heterogeneous population. However, the key innovation of this study lies in transcending aggregate comparisons by modeling individualized treatment effects.^9^, ^10^, ^12^, ^16^, ^17^, ^20^

Through its counterfactual treatment estimation architecture, the DUSK tool enables an estimation of how a given patient might fare with EVT versus MM, accounting for both baseline risk and potential treatment effect heterogeneity. This individualized framework revealed that among patients with moderate to severe strokes (NIHSS ≥ 8), EVT was associated with a significantly increased likelihood of achieving excellent functional outcome at 90 days (aOR = 3.00, 95% CI = 1.69–5.32; *P*_interaction_ = 0.001), even after Bonferroni correction. A similar trend toward benefit was also observed in subgroup analyses of recent RCTs.^6^, ^10^, ^17^ This effect modification by stroke severity, captured within the DUSK tool among other features, illustrates the importance of tailoring treatment based on both clinical and imaging biomarkers, not average effects. The external validation of our model using an independent real‐world cohort confirmed its robustness and generalizability. The model, despite the small sample size, demonstrated stable discriminative performance, acceptable calibration, and a consistent ability to detect favorable outcomes, particularly in EVT‐treated patients with moderate‐to‐severe stroke presentations. Importantly, the validation cohort included a higher proportion of patients with more severe deficits (median NIHSS: 13 in the EVT group), contrasting with the selection patterns of recent randomized trials. Compared with the HERMES meta‐analysis, which predominantly included patients with LVOs, recent DMVO trials (DISCOUNT, DISTAL, and ESCAPE‐MeVO) enrolled substantially older patients (median age = ~74–77 vs. 68 in HERMES) and those with lower stroke severity (median NIHSS 6–8 vs. 17 overall, and 14–15 in the M2 subgroup).^3^, ^6^, ^10^, ^17^, ^39^ Whereas IVT use was slightly lower in the DMVO trials (57–71%) compared to HERMES (85%), this remains a key consideration, as IVT may be more effective in DMVOs due to smaller clot burden and more distal locations. Despite differences in patient profiles, successful reperfusion rates were comparable or even slightly higher in DMVO trials (72–77%) versus HERMES (71%), suggesting that EVT can achieve technical success in appropriately selected DMVO cases.^3^, ^6^, ^10^, ^17^ Such selection bias limits the generalizability of their findings to broader DMVO populations, particularly those with moderate or severe deficits who are more commonly encountered in clinical practice.^16^ Our model, developed and validated using data from more heterogeneous and clinically representative populations, may therefore offer complementary insight, especially in settings where rigid trial inclusion criteria may not apply. Several ongoing trials are investigating EVT for DMVOs across different regions and designs. The ORIENTAL‐MEVO trial in China enrolled 564 patients with co‐dominant or non‐dominant M2/M3, A1–A3, or P1–P3 occlusions within a 24‐hour window, requiring NIHSS ≥ 6 and allowing IVT, with mRS shift as the primary outcome while focusing on an Asian population. The DISTALS trial (United States and the European Union) included 118 patients with similar target occlusions within 24 hours, excluded IVT, enrolled NIHSS 2 to 24, and focused on successful reperfusion without symptomatic ICH, using a novel radially adjustable stent retriever. The DUSK trial (United States and the European Union) enrolled 564 patients with the same occlusion targets within 12 hours, specifically including patients who failed i.v. lytic therapy and had disabling deficits, with mRS shift as the primary end point. The STEP trial in the United States is enrolling 600 patients with M2/M3 occlusions within 24 hours, requires NIHSS ≥ 8, allows IVT, and uses utility‐weighted mRS (UW‐mRS) as the outcome, using an adaptive enrichment Bayesian design. The Imperative trial (United States) appears to focus on M2 occlusions—possibly including dominant M2s and uses Zoom aspiration technology, with mRS as the outcome, although limited data are available. Similarly, the Penumbra trial (United States) targets DMVOs using Penumbra aspiration devices, but details on sample size, inclusion criteria, and outcomes are not yet disclosed.

The strength of the DUSK tool lies in its integration of domain‐relevant binary features (eg, dichotomized NIHSS, age, and glucose) and interpretable interaction terms (eg, NIHSS × treatment and time × treatment) informed by clinical heuristics and pathophysiology. Feature selection using forward and backward algorithms refined these models to maximize predictive accuracy while maintaining interpretability. Internal validation via bootstrapping yielded robust metrics, and, importantly, external validation in a separate real‐world cohort confirmed generalizability. These findings validate the DUSK tool as a reliable option to support shared decision making in the absence of RCT data. This precision modeling approach challenges the conventional binary treatment paradigm and instead offers a granular probability‐based framework. For instance, 2 patients with identical occlusion location may differ substantially in their modeled benefit from EVT depending on factors such as infarct volume, glucose level, or delay to imaging. In this regard, DUSK aligns with a broader shift in stroke care toward individualized medicine by translating population‐level patterns into actionable, patient‐specific insights.

This study has several limitations. The retrospective, nonrandomized nature of the multicenter cohort introduces inherent selection bias and unmeasured confounding, such as operator expertise or anatomic complexity, which may influence both treatment assignment and outcomes. Although we implemented IPTW and validated findings against a causal T‐Learner, these associations derived from observational data do not establish definitive causality. Consequently, the DUSK tool provides estimated probabilities of favorable outcomes that require prospective validation in a randomized setting to confirm the causal nature of the treatment effects. Imaging adjudication was performed locally at each participating center rather than by a central core laboratory, potentially introducing variability or misclassification bias in occlusion site classification, particularly at vessel segment junctions. Furthermore, CTP metrics, such as ischemic core and penumbral volumes, were excluded due to high missingness rates and protocol variability. Given that favorable perfusion profiles and penumbral salvage are strong predictors of functional recovery in medium vessel occlusions, future iterations of the tool should incorporate standardized perfusion data when available. Additionally, our analysis did not capture cognitive or vision‐specific outcomes, which are particularly relevant for posterior circulation strokes.

Despite these limitations, this study represents a methodologically rigorous effort to move beyond aggregate binary comparisons toward a patient‐level, interpretable clinical framework. The DUSK tool empowers clinicians to transition from anecdotal decision making to data‐driven, individualized care strategies for a challenging stroke population.

In conclusion, although EVT did not demonstrate universal benefit over MM in this cohort, the DUSK tool identified that EVT may confer substantial benefit in patients with moderate to severe strokes. The application of explainable counterfactual treatment estimation, informed by domain‐specific variables and validated externally, offers a transformative approach to personalized stroke care. By bridging the gap between clinical uncertainty and action, the DUSK tool helps individualize treatment in one of the most heterogeneous and controversial domains of acute stroke intervention.

## Author Contributions

M.F.D. and R.G.N. contributed to the conception and design of the study; M.F.D., M.H.M., H.A., M.O.G., M.R.G., M.R., J.T.F., J.E.S., S.O.G., M.A., A.A., W.H., T.N.N., and D.C.H. contributed to the acquisition and analysis of data; M.F.D., M.H.M., and R.G.N. contributed to drafting the text and preparing the figures.

## Potential Conflicts of Interest

R.G.N. reports consultant compensation from Anaconda Biomed, Corindus Inc, Genentech, Vesalio, Imperative Care, Biogen Inc, Cerenovus, RapidPulse, Medtronic USA Inc, Prolong Pharmaceuticals, Perfuze, Ceretrieve, Phenox, Brainomix, Stryker Corporation, NeuroVasc Technologies Inc, and Viz‐AI, and holds stock options in Brainomix, Ceretrieve, Viz‐AI, Vesalio, Corindus Inc, and Perfuze. D.C.H. reports stock options in Viz‐AI and consultant compensation from Cerenovus, Stryker, Vesalio, Brainomix, Chiesi USA Inc, and Poseydon Medical, as well as compensation from the Jacobs Institute for data and safety monitoring services. T.N.N. reports research support from Brainomix and a relationship with Idorsia. S.O.G. reports consulting fees for advisory roles with Stryker Neurovascular, Medtronic, and MicroVention, and research support from Medtronic, Carver College of Medicine–University of Iowa, methinks, the National Institutes of Health, Siemens, Stryker, and MicroVention. J.E.S. reports consultant compensation from Ceribell and other compensation from AstraZeneca. A.A. reports consultant compensation from Johnson & Johnson. J.T.F. reports compensation from MIVI for data and safety monitoring services and consultant compensation from Stryker Corporation, MicroVention Inc, Penumbra Inc, Cerenovus, and Viz‐AI, and holds stock in Imperative Care. The other authors have nothing to report.

## Supporting information


**Supplementary Figure S1.** Crude distribution of 90‐day modified Rankin scale (mRS) scores in Distal and Medium Vessel Occlusion Stroke (DUSK).
**Supplementary Figure S2.** Model feature coefficients. Horizontal bar chart displaying the log‐odds coefficients for all predictors in the Penalized Logistic Regression (Elastic Net) model. Dark blue bars represent core/fixed features retained a priori based on clinical rationale. Light blue bars represent features selected by the Elastic Net regularization process. Features are sorted by absolute coefficient magnitude. National Institutes of Health Stroke Scale (NIHSS) ≥ 8 shows the largest negative coefficient (−1.05), indicating strong association with unfavorable outcomes. The Treatment × NIHSS interaction term shows the largest positive coefficient among interaction terms (0.41), suggesting differential endovascular therapy (EVT) benefit in patients with higher stroke severity. Coefficients represent the change in log‐odds of favorable outcome (mRS 0–2) per unit change in the predictor, holding other variables constant.
**Supplementary Table S1.** Model performance comparison (5000‐fold cross‐validation).
**Supplementary Table S2.** Validation of heterogeneity of treatment effect–internal cohort (comparison with causal T‐learner).
**Supplementary Table S3.** Characteristics of external validation cohort.
**Supplementary Table S4.** External validation performance (independent cohort, n = 86).
**Supplementary Table S5.** Validation of heterogeneity of treatment effect – external cohort.

## Data Availability

The data that support the findings of this study are available from the corresponding and leading authors upon reasonable request.
