# Supplementary material for: Individualized Treatment in Distal and Medium Vessel Occlusion Stroke Using a Validated Explainable Counterfactual Treatment Estimation Model
Source: Ann Neurol. 2026 Jan 28;99(5):1198–209. doi: 10.1002/ana.78168 (PMC13092792; doi:10.1002/ana.78168)
Supplement: Supplementary file 1 — Supplementary Figure S1. Crude distribution of 90‐day modified Rankin scale (mRS) scores in Distal and Medium Vessel Occlusion Stroke (DUSK). Supplementary Figure S2. Model feature coefficients. Horizontal bar chart displaying the log‐odds coefficients for all predictors in the Penalized Logistic Regression (Elastic Net) model. Dark blue bars represent core/fixed features retained a priori based on clinical rationale. Light blue bars represent features selected by the Elastic Net regularization process. Features are sorted by absolute coefficient magnitude. National Institutes of Health Stroke Scale (NIHSS) ≥ 8 shows the largest negative coefficient (−1.05), indicating strong association with unfavorable outcomes. The Treatment × NIHSS interaction term shows the largest positive coefficient among interaction terms (0.41), suggesting differential endovascular therapy (EVT) benefit in patients with higher stroke severity. Coefficients represent the change in log‐odds of favorable outcome (mRS 0–2) per unit change in the predictor, holding other variables constant. Supplementary Table S1. Model performance comparison (5000‐fold cross‐validation). Supplementary Table S2. Validation of heterogeneity of treatment effect–internal cohort (comparison with causal T‐learner). Supplementary Table S3. Characteristics of external validation cohort. Supplementary Table S4. External validation performance (independent cohort, n = 86). Supplementary Table S5. Validation of heterogeneity of treatment effect – external cohort. [file ANA-99-1198-s001.docx]

SUPPLEMENTARY

**Figure S1.** Crude Distribution of 90-Day Modified Rankin Scale (mRS) Scores in DUSK Cohort

**Figure S2.** Model Feature Coefficients.

**Table S1.** Model Performance Comparison (5000-fold Cross-validation)

**Table S2.** Validation of Heterogeneity of Treatment Effect – Internal Cohort (Comparison with Causal T-Learner)

**Table S3.** Characteristics of external validation cohort

**Table S4.** External Validation Performance (Independent Cohort, n = 86)

**Table S5.** Validation of Heterogeneity of Treatment Effect – External Cohort (Comparison with Causal T-Learner)


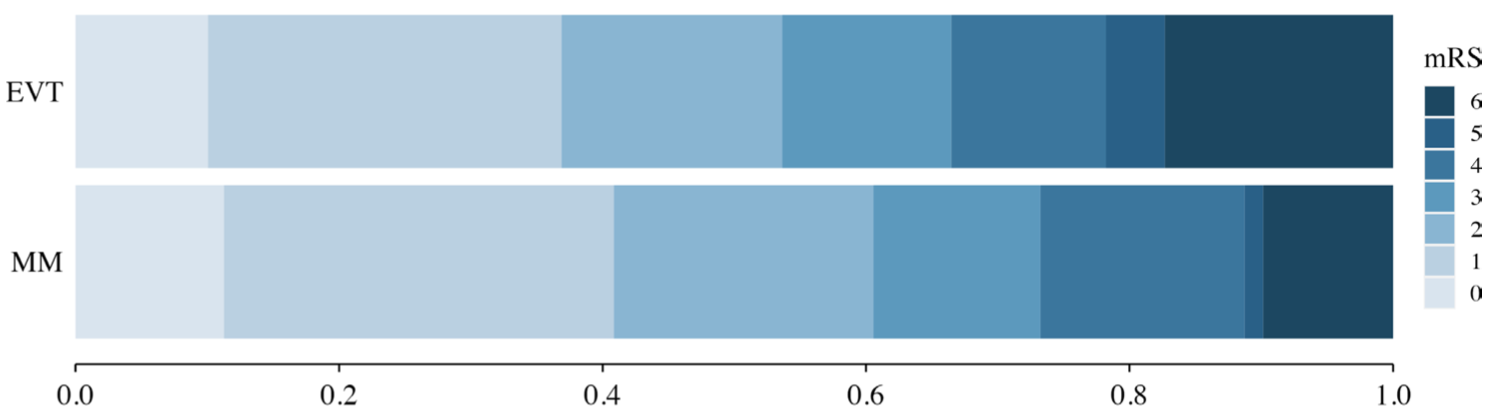


**Figure S1. Crude Distribution of 90-Day Modified Rankin Scale (mRS) Scores in DUSK**

**Cohort**

**
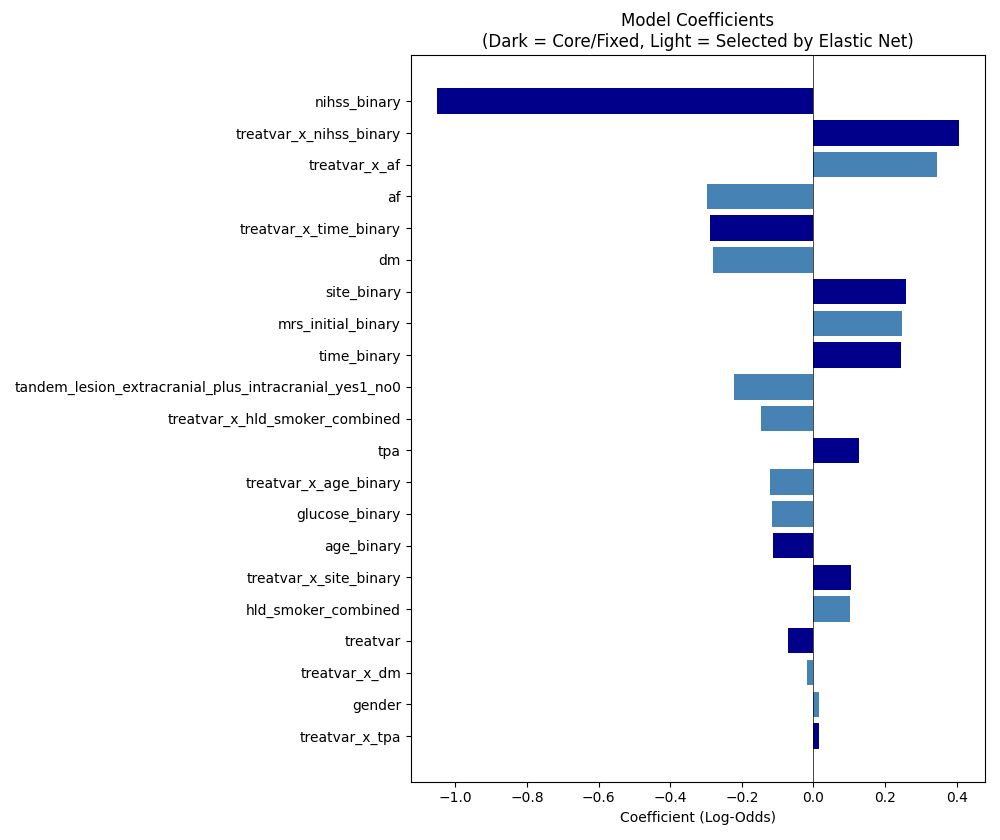
**

**Figure S2. Model Feature Coefficients.** Horizontal bar chart displaying the log-odds coefficients for all predictors in the Penalized Logistic Regression (Elastic Net) model. Dark blue bars represent core/fixed features retained a priori based on clinical rationale. Light blue bars represent features selected by the Elastic Net regularization process. Features are sorted by absolute coefficient magnitude. NIHSS ≥ 8 shows the largest negative coefficient (-1.05), indicating strong association with unfavorable outcomes. The Treatment × NIHSS interaction term shows the largest positive coefficient among interaction terms (0.41), suggesting differential EVT benefit in patients with higher stroke severity. Coefficients represent the change in log-odds of favorable outcome (mRS 0-2) per unit change in the predictor, holding other variables constant.

**Table S1. Model Performance Comparison (****5000-fold Cross-validation)**

| Model | AUC | Brier Score | Sensitivity | Accuracy |
| --- | --- | --- | --- | --- |
| Logistic Regression | 0.72 | 0.21 | 0.73 | 0.68 |
| Decision Tree Classifier | 0.69 | 0.25 | 0.67 | 0.64 |
| SVC Linear | 0.69 | 0.23 | 0.65 | 0.63 |
| XGBoost | 0.68 | 0.24 | 0.71 | 0.63 |

Table S2. Validation of Heterogeneity of Treatment Effect – Internal Cohort (Comparison with Causal T-Learner)

| Comparison Metric | Value | Interpretation |
| --- | --- | --- |
| Pearson Correlation (r) | 0.968 | Extremely high correlation between models |
| Treatment Agreement Rate | 92.5% | Models recommend same treatment for 92.5% of patients |
| Mean Difference in CATE | 0.0004 | Negligible systematic bias between models |
| Interaction Model Mean CATE (SD) | 0.021 (0.132) | Average predicted EVT benefit ~2.1% |
| T-Learner Mean CATE (SD) | 0.020 (0.135) | Benchmark causal model estimates similar benefit |

Abbreviations: CATE, Conditional Average Treatment Effect; SD, standard deviation. Note: The high correlation (r = 0.97) and agreement rate (92.5%) validate the use of the Penalized Logistic Regression model for personalized treatment recommendations, as it closely approximates a sophisticated causal inference model.

**Table S3. Characteristics of external validation cohort**

| Variable | Overall (n=86) | MM (n=37) | EVT (n=49) | P-Value |
| --- | --- | --- | --- | --- |
| Diabetes mellitus | 20 (23.3%) | 6 (16.2%) | 14 (28.6%) | 0.278 |
| Atrial fibrillation | 15 (17.4%) | 3 (8.1%) | 12 (24.5%) | 0.090 |
| Female gender | 52 (60.5%) | 23 (62.2%) | 29 (59.2%) | 0.955 |
| tPA administration | 60 (69.8%) | 37 (100.0%) | 23 (46.9%) | <0.001 |
| Hyperlipidemia | 53 (61.6%) | 24 (64.9%) | 29 (59.2%) | 0.755 |
| Smoker | 16 (18.6%) | 6 (16.2%) | 10 (20.4%) | 0.830 |
| Age > cutoff | 18 (20.9%) | 6 (16.2%) | 12 (24.5%) | 0.505 |
| NIHSS > cutoff | 57 (66.3%) | 12 (32.4%) | 45 (91.8%) | <0.001 |
| High glucose | 9 (10.5%) | 3 (8.1%) | 6 (12.2%) | 0.726 |
| Baseline mRS > cutoff | 78 (90.7%) | 36 (97.3%) | 42 (85.7%) | 0.130 |
| Time > cutoff | 65 (75.6%) | 32 (86.5%) | 33 (67.3%) | 0.073 |
| HLD & smoker combined | 61 (70.9%) | 27 (73.0%) | 34 (69.4%) | 0.902 |
| Site (anterior) | 62 (72.1%) | 25 (67.6%) | 37 (75.5%) | 0.569 |
| Age (median [Q1,Q3]) | 72.0 [61.0,78.8] | 68.0 [60.0,76.0] | 75.0 [63.0,80.0] | 0.164 |
| NIHSS (median [Q1,Q3]) | 10.0 [6.0,16.8] | 6.0 [4.0,10.0] | 13.0 [10.0,20.0] | <0.001 |
| Glucose (median [Q1,Q3]) | 117.0 [101.0,142.2] | 114.5 [98.8,139.0] | 119.5 [103.8,149.8] | 0.364 |
| Baseline mRS (median [Q1,Q3]) | 0.0 [0.0,1.0] | 0.0 [0.0,0.0] | 0.0 [0.0,1.0] | 0.183 |
| Time (median [Q1,Q3]) | 265.0 [166.0,345.0] | 203.0 [150.0,315.0] | 280.0 [198.0,444.0] | 0.033 |

Table S4. External Validation Performance (Independent Cohort, n = 86)

| Performance Metric | Mean Value | Standard Deviation | 95% Confidence Interval |
| --- | --- | --- | --- |
| AUC (Discrimination) | 0.738 | 0.053 | 0.628 – 0.835 |
| Brier Score (Calibration) | 0.211 | 0.022 | 0.169 – 0.254 |
| Accuracy | 67.5% | 5.0% | 58.1% – 76.7% |
| Sensitivity | 76.6% | 6.1% | 64.4% – 88.2% |
| Specificity | 56.4% | 8.0% | 40.0% – 71.4% |
| Precision (PPV) | 68.1% | 6.3% | 55.8% – 79.6% |
| NPV | 66.4% | 8.3% | 50.0% – 82.8% |
| F1 Score | 0.719 | 0.050 | 0.617 – 0.811 |

Abbreviations: AUC, area under the receiver operating characteristic curve; PPV, positive predictive value; NPV, negative predictive value. Note: Results based on 1,000 bootstrap iterations. The model maintains robust discrimination (AUC 0.74) and sensitivity (76.6%) in an independent population, confirming generalizability.

Table S5. Validation of Heterogeneity of Treatment Effect – External Cohort

| Comparison Metric | Value | Interpretation |
| --- | --- | --- |
| Pearson Correlation (r) | 0.976 | Extremely high correlation maintained externally |
| Treatment Agreement Rate | 89.5% | Models recommend same treatment for 89.5% of patients |
| Mean Difference in CATE | -0.0004 | Negligible systematic bias between models |
| Interaction Model Mean CATE (SD) | 0.015 (0.125) | Average predicted EVT benefit ~1.5% |
| T-Learner Mean CATE (SD) | 0.015 (0.127) | Consistent with internal validation findings |

Abbreviations: CATE, Conditional Average Treatment Effect; SD, standard deviation. Note: External validation confirms the robustness of treatment effect heterogeneity estimates, with correlation (r = 0.98) even higher than internal validation.
